# Murine and Human Myogenic Cells Identified by Elevated Aldehyde Dehydrogenase Activity: Implications for Muscle Regeneration and Repair

**DOI:** 10.1371/journal.pone.0029226

**Published:** 2011-12-15

**Authors:** Joseph B. Vella, Seth D. Thompson, Mark J. Bucsek, Minjung Song, Johnny Huard

**Affiliations:** 1 Department of Orthopedic Surgery, Stem Cell Research Center, Children's Hospital of Pittsburgh, Pittsburgh, Pennsylvania, United States of America; 2 Department of Bioengineering, University of Pittsburgh, Pittsburgh, Pennsylvania, United States of America; 3 McGowen Institute of Regenerative Medicine, University of Pittsburgh, Pittsburgh, Pennsylvania, United States of America; University of Medicine and Dentistry of New Jersey, United States of America

## Abstract

**Background:**

Despite the initial promise of myoblast transfer therapy to restore dystrophin in Duchenne muscular dystrophy patients, clinical efficacy has been limited, primarily by poor cell survival post-transplantation. Murine muscle derived stem cells (MDSCs) isolated from slowly adhering cells (SACs) via the preplate technique, induce greater muscle regeneration than murine myoblasts, primarily due to improved post-transplantation survival, which is conferred by their increased stress resistance capacity. Aldehyde dehydrogenase (ALDH) represents a family of enzymes with important morphogenic as well as oxidative damage mitigating roles and has been found to be a marker of stem cells in both normal and malignant tissue. In this study, we hypothesized that elevated ALDH levels could identify murine and human muscle derived cell (hMDC) progenitors, endowed with enhanced stress resistance and muscle regeneration capacity.

**Methodology/Principal Findings:**

Skeletal muscle progenitors were isolated from murine and human skeletal muscle by a modified preplate technique and unfractionated enzymatic digestion, respectively. ALDH^hi^ subpopulations isolated by fluorescence activate cell sorting demonstrated increased proliferation and myogenic differentiation capacities compared to their ALDH^lo^ counterparts when cultivated in oxidative and inflammatory stress media conditions. This behavior correlated with increased intracellular levels of reduced glutathione and superoxide dismutase. ALDH^hi^ murine myoblasts were observed to exhibit an increased muscle regenerative potential compared to ALDH^lo^ myoblasts, undergo multipotent differentiation (osteogenic and chondrogenic), and were found predominately in the SAC fraction, characteristics that are also observed in murine MDSCs. Likewise, human ALDH^hi^ hMDCs demonstrated superior muscle regenerative capacity compared to ALDH^lo^ hMDCs.

**Conclusions:**

The methodology of isolating myogenic cells on the basis of elevated ALDH activity yielded cells with increased stress resistance, a behavior that conferred increased regenerative capacity of dystrophic murine skeletal muscle. This result demonstrates the critical role of stress resistance in myogenic cell therapy as well as confirms the role of ALDH as a marker for rapid isolation of murine and human myogenic progenitors for cell therapy.

## Introduction

Duchenne muscular dystrophy is a degenerative muscle disease caused by a mutation of the gene encoding dystrophin, a protein that anchors the myofiber cytoskeleton to the basal lamina, resulting in muscle fiber necrosis and progressive weakness [1], [2]. Despite extensive investigation of various approaches to deliver dystrophin to dystrophic muscle, few treatment options for patients with this devastating disease exist [3], [4]. Myoblast transfer therapy, defined as the transplantation of normal myoblasts into dystrophin-deficient muscle, has been shown to transiently deliver dystrophin to dystrophic myofibers as well as improve muscle contraction force [5]. However outcomes of this approach are limited by immune rejection, limited cell migration with the formation of cell pockets, and poor cell survival rates, which is perhaps the most important barrier to efficacious myogenic cell therapy [6], [7], [8]. Pursuit of novel myogenic progenitors and delivery approaches that would mitigate this cell loss are active areas of research [9], [10], [11], [12].

Numerous myogenic progenitors have been isolated from post-natal murine and human skeletal muscle for cell therapy such as satellite cells, myoblasts, MDSCs, side-population cells, Sk-DN/Sk-34 cells, pericytes, mesangioblasts, human SMALD^+^ cells, and myo-endothelial cells [5], [13], [14], [15], [16]. Some of these myogenic cell types have demonstrated excellent muscle regeneration capacities in vivo; however, in our experience the common behavior of myogenic progenitors that induce robust muscle regeneration is their increased capacity to withstand oxidative and inflammatory stress [9], [10], [11]. The muscle derived stem cell (MDSC), a myogenic progenitor isolated from the slowly adhering fraction of the preplate technique, has been shown to induce greater skeletal muscle regeneration than myoblasts largely due to their increased capacity to resist oxidative stress [9], [17]. This stress resistance capacity is necessary to survive, proliferate, and differentiate under conditions of inflammation, an environment of oxidative and inflammatory stress that causes a precipitous loss in transplanted cell viability [18], [19], [20], [21].

Previously we demonstrated the central role that the intracellular antioxidant glutathione (GSH) plays in the increased survival and muscle regenerative capacity of MDSCs. Increased levels of GSH in MDSCs compared to myoblasts was correlated with the increased rates of survival, proliferation, and myogenic differentiation in conditions of oxidative and inflammatory stress [9]. When the GSH levels of MDSCs were reduced using diethyl maleate (DEM) to levels that are observed in myoblasts, a significant reduction in the ability of MDSCs to regenerate skeletal and cardiac muscle was observed [9]. In fact, the regeneration capacity of the MDSCs with diminished GSH levels was statistically equivalent to that observed in myoblasts. In contrast, by increasing the GSH levels of MDSCs using n-acetylcysteine treatment, the cardiac and skeletal muscle regeneration was significantly improved compared to untreated MDSCs [11]. These studies led us to hypothesize that the muscle regenerative capacity of a myogenic cell is primarily determined by its capacity to withstand oxidative and inflammatory stress, rather than the extent of its stem cell-like characteristics such as self-renewal and multilineage differentiation potential. In fact, other groups have suggested that increased stress resistance may be a primary characteristic of stemness for a variety of stem cell populations [22], [23], [24], [25].

In the current study, we sought to further validate this hypothesis through the isolation of myogenic progenitors with enhanced stress resistance using elevated expression of cytosolic aldehyde dehydrogenase (ALDH1A1) as a marker for this trait. ALDH represents a family of intracellular enzymes that regulate retinoic acid (RA) concentration, which plays an important role in embryonic myogenesis, by driving the expression of multiple myogenic regulatory factors (MRFs) in murine and human embryonic stem cells [26], [27], [28], [29]. ALDH activity has been used to identify and sort primitive progenitors from multiple tissues [30]. Elevated aldehyde dehydrogenase (ALDH) has been shown to be a marker of progenitor status in hematopoietic [31], [32], mesenchymal [33], endothelial [34], neural [35], [36], and recently human skeletal muscle cell populations [16], [37], as well as being a marker of cancer stem cells or metastasis competent tumor cells [38]. Perhaps more importantly, ALDH activity has been shown to directly mitigate oxidative damage by converting aldehyde by-products of lipid peroxidation to non-reactive carboxylic acids and has been associated with other mechanisms of increased antioxidant capacity in somatic and cancer stem cells [39], [40], [41]. For example, hematopoietic stem cells present upregulated FoxO (Forkhead O) transcription factors, downstream targets of the PI3K-AKT pathway and regulators of the oxidative stress response, which include induction of MnSOD, catalase, and GADD45 [42], [43]. Endothelial progenitors isolated from umbilical cord blood present elevated MnSOD levels that significantly increase their oxidative stress resistance and effectiveness in treating tissue ischemia [44], [45]. Similar properties of increased expression of free radical scavenging systems and decreased levels of reactive oxygen species is observed in epithelial breast cancer stem cells [46], [47], [48].

Subpopulations of skeletal muscle derived cells that expressed high and low levels of ALDH (ALDH^hi^ and ALDH^lo^) were isolated from cultured murine and human skeletal muscle by fluorescence activated cell sorting (FACS) as depicted in Figure S1. These ALDH^lo^ and ALDH^hi^ subpopulations were isolated from preplate derived murine myoblasts and MDSCs in addition to unfractionated human muscle derived cells (hMDCs) [12]. The capacity of ALDH^lo^ and ALDH^hi^ populations to withstand oxidative and inflammatory stress conditions, in terms of proliferation and myogenic differentiation, was examined in addition to their skeletal muscle regeneration capacity in vivo. This stress resistance capacity was then correlated with intracellular antioxidant levels, in the form of GSH and superoxide dismutase (SOD). Observations of increased ALDH activity in human muscle derived cells have been made previously by Jean et al. and Vauchez et al., who demonstrated the myogenic capacity of these subpopulations both in vitro and in vivo [16], [37]. Jean et al. also observed an increased survival capacity of ALDH^hi^ human myoblasts when exposed to H_2_O_2_ in vitro, yet found no analogous ALDH^hi^ subpopulation in murine muscle isolates [37]. However, differences in our observations may be attributed to differences in our isolation protocol, which is addressed in the discussion section. Further, we studied the multilineage differentiation potential of murine ALDH^hi^ myoblasts in vitro, by comparing the osteogenic and chondrogenic potential of ALDH^hi^ to that of ALDH^lo^ myoblasts. A summary of the findings described in the present study can be found in the Table S1. Our results suggest that increased ALDH activity identifies myogenic progenitors in both murine and human skeletal muscle with increased oxidative and inflammatory stress resistance capacity. Furthermore, these results highlight the important role of stress resistance in stem cell mediated muscle regenerative therapies.

## Results

### Isolation of ALDH^lo^ and ALDH^hi^ populations of skeletal muscle derived cells

Cells with elevated ALDH levels become fluorescent when exposed to boron-dipyrromethene (BODIPY) labeled amino acetaldehyde (Aldefluor, StemCell Technologies) and can be isolated using fluorescence activated cell sorting (FACS). The non-polar BODIPY-aminoacetaldehyde diffuses freely into the cytoplasm and is converted by cytosolic ALDH1A1 to the negatively charged BODIPY-aminoacetate, which accumulates in the cytoplasm and causes the cell to fluoresce with an emission peak at 513 nm [49]. Cells, of low side scatter, whose brightness exceeded the gated intensity of the diethylaminobenzaldehyde (DEAB) inhibited population were deemed ALDH^hi^ and isolated from heterogeneous populations of murine and human muscle derived cells.

**Figure 1:**
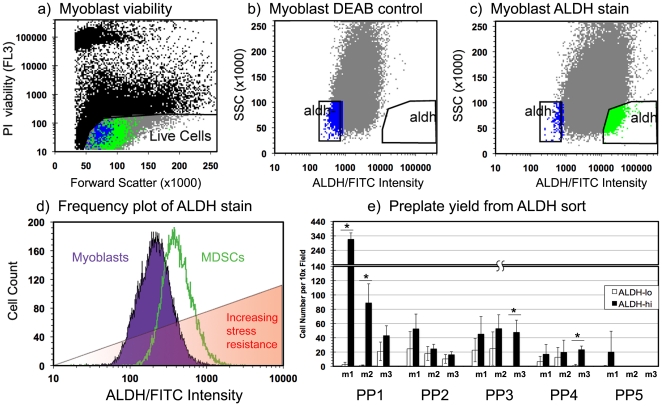
Fluorescence activated cell sorting (FACS) of muscle derived cells by aldehyde dehydrogenase activity. Isolation of ALDH^lo^ and ALDH^hi^ subpopulations from cultured myoblasts was performed using FACS. (a) Dead cells were excluded from the isolation by detection of nuclear propidium iodide fluorescence. (b, c) ALDH^hi^ SCC^lo^ (high ALDH activity, low side scatter) cells were isolated from a heterogeneous population of myoblasts using DEAB, a potent inhibitor of ALDH, as a gating control. (d) Measurement of the FITC channel signal intensity of Aldefluor stained murine myoblasts and MDSCs demonstrated a shift in the distribution of signal intensity between the two populations, suggesting an increase in the median ALDH activity in MDSCs compared to myoblasts. (e) ALDH sorted murine muscle derived cells were preplated to demonstrate the increased yield of cells in later preplate cycles from ALDH^hi^ cells (up to PP5) compared to ALDH^lo^ cells. Cells were isolated from three mice labeled m1, m2, and m3. (* indicates p<0.05).

ALDH^hi^ cell subpopulations were isolated from rapidly adhering preplate myoblasts, slowly adhering MDSCs as well as unfractionated hMDCs [12]. Figure 1 illustrates a representative FACS isolation of ALDH^hi^ and ALDH^lo^ subpopulations of murine myoblasts. Dead cells were excluded from FACS isolations by detection of nuclear propidium iodide staining (Figure 1a). The gating for ALDH^hi^ and ALDH^lo^ cell isolations were determined by ALDH fluorescence extremes of DEAB inhibited controls (and low side scatter) of each population as illustrated in Figure 1b. ALDH^lo^ and ALDH^hi^ myoblasts represented roughly 1–5% of the total myoblast population (Figure 1c).

We observed an elevated median Aldefluor fluorescence in murine MDSCs compared to myoblasts, despite the absence of a difference in autofluorescence in their untreated controls (data not shown). In addition, MDSCs appear to have a more homogenous elevated ALDH activity than myoblasts, indicating less heterogeneity in ALDH expression. The red overlay in Figure 1d indicates a trend of increased stress resistance observed in MDSCs compared to myoblasts and is intended to emphasize the increase in stress resistance that we observed in cells with elevated ALDH activity. It should also be noted that we typically observed increased cell viability in ALDH^hi^ cells compared to ALDH^lo^ cells immediately following FACS isolation (data not shown). That is, the mechanical stress of the cell segregation process of flow cytometry typically had a more deleterious effect on the ALDH^lo^ cell viability compared to the ALDH^hi^ cells.

To verify this trend of increased ALDH activity in slowly adhering MDSCs, the preplate technique was performed using ALDH^lo^ and ALDH^hi^ cells isolated from dissociated murine skeletal muscle (Figure 1e). A trend of increased numbers of preplate SACs (PP3 and beyond) was obtained by preplating ALDH^hi^ cells, when compared to performing the preplate technique on ALDH^lo^ cells. However it is possible that fewer SACs were obtained from the ALDH^lo^ subpopulation, compared to the ALDH^hi^ subpopulation simply due to the decreased viability of ALDH^lo^ cells following the cumulative cellular damage of enzymatic digestion and FACS isolation. We attribute the paucity of cells in preplate populations PP5 and beyond to these stressors in addition to the necessary delay in initiating the preplate process. However it should be noted that variability in late preplate yield (PP5 and PP6) has been observed in previous studies despite the consistent observation of enrichment in the MDSC population in successive preplate cycles beyond PP2 [12].

### Increased stress resistance, proliferation, differentiation and muscle regeneration capacity of ALDH^hi^ sorted murine myoblasts

**Figure 2:**
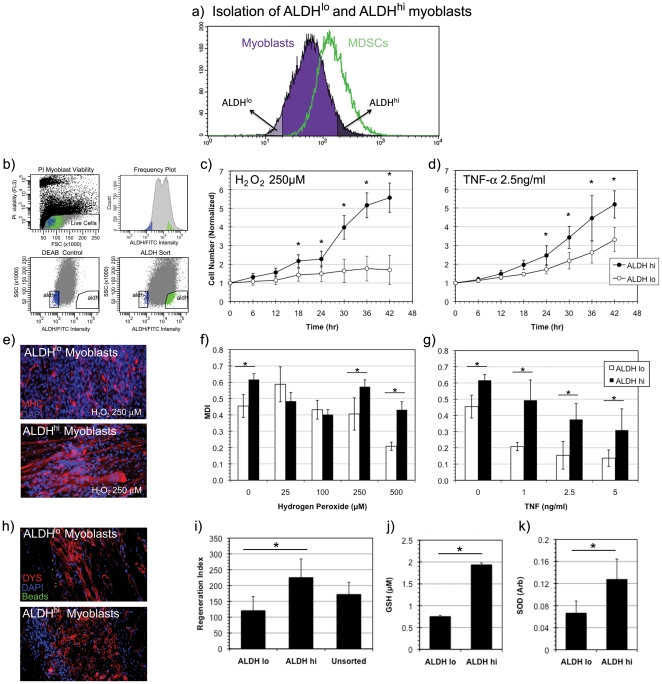
Isolation and stress resistance capacity of ALDH^hi^ myoblasts. (a, b) ALDH^hi^ murine myoblasts were isolated via fluorescence activated cell sorting (FACS) from a heterogeneous population of skeletal muscle derived cells using DEAB, a potent inhibitor of ALDH, as a gating control. (c, d) A significantly increased rate of proliferation of ALDH^hi^ myoblasts compared to ALDH^lo^ myoblasts was observed in conditions of oxidative stress (H_2_O_2_, 250 µM) (* indicates p<0.05) and inflammatory stress conditions (TNF-α, 2.5 ng/ml) (n = 9). (e) ALDH^lo^ and ALDH^hi^ myoblasts underwent myogenic differentiation by fusing into MHC+ myotubes (red) under oxidative stress conditions (H_2_O_2_, 250 µM). Nuclei were stained with DAPI (blue). (f) Significantly increased myogenic differentiation indices (MDI) were observed in ALDH^hi^ cells at several concentrations of oxidative stress (0, 250 and 500 µM) when compared to ALDH^lo^ (n = 3). (g) Similarly increased MDIs of ALDH^hi^ cells were observed under all inflammatory stress conditions when compared to ALDH^lo^ myoblasts. (h) An increased number and density of dystrophin positive myofibers (stained in red) were observed in *mdx* mice injected intramuscularly with ALDH^hi^ cells compared to those injected with ALDH^lo^ myoblasts. Nuclei are DAPI (blue) stained. Green FluoSphere beads are observed to localize at the area of initial injection. (i) A significantly increased regeneration index was observed in ALDH^hi^ transplanted mice compared to those injected with ALDH^lo^ and unsorted cells. (n = 3) (j, k) Measurements of intracellular antioxidants glutathione (GSH) and superoxide dismutase (SOD) levels demonstrated statistically significant elevation of both antioxidants in ALDH^hi^ myoblasts compared to ALDH^lo^ myoblasts (n = 3).

Myoblasts isolated from rapidly adhering cells (RACs) using the preplate technique have been previously characterized as a heterogeneous population in various states of quiescence, activation, and differentiation [17], [50], [51]. We therefore hypothesized that we may also observe heterogeneity in their ALDH expression. ALDH^lo^ and ALDH^hi^ subpopulations of murine myoblasts were isolated by FACS as depicted in Figure 2a. FACS gating was set using DEAB inhibition of ALDH activity as described previously and illustrated in Figure 2b.

Following FACS isolation of ALDH^lo^ and ALDH^hi^ subpopulations from the RACs, we quantified the oxidative (hydrogen peroxide or H_2_O_2_) and inflammatory (TNF-α) stress resistance of these subpopulations during proliferation and myogenic differentiation [52]. Hydrogen peroxide is a strong oxidant that is formed by the dismutation of superoxide and freely diffuses across the cell membrane. TNF-α on the other hand is an acute phase cytokine that has been shown to inhibit myogenic differentiation via NF-kB induction, promote caspase mediated apoptosis, and induce reactive oxygen species accumulation in mitochondria [53], [54].

Significant differences in stress resistance capacity were observed between ALDH^lo^ and ALDH^hi^ subpopulations of murine myoblasts in terms of proliferation and myogenic differentiation. The quantification of proliferation rates of ALDH^lo^ and ALDH^hi^ myoblasts was performed using live cell imaging microscopy in conditions of oxidative and inflammatory stress. This study showed a significant proliferation advantage by the ALDH^hi^ cells compared to ALDH^lo^ cells (Figure 2c,d). The myogenic differentiation capacity was quantified using the myogenic differentiation index (MDI), a measure of the ratio of nuclei in myosin heavy chain (MHC) expressing myofibers to total number of nuclei as defined previously [55]. Myogenic differentiation of ALDH^lo^ and ALDH^hi^ myoblasts was induced by a 4d exposure to low serum (2% horse serum) differentiation medium (DM) conditions in the presence of oxidative and inflammatory stress (Figure 2e-g). A significantly increased proportion of MHC expressing cells was observed in ALDH^hi^ myoblasts compared to ALDH^lo^ myoblasts (Figure 2e-g), indicating that ALDH^hi^ myoblasts not only preserve their competence for proliferation but also differentiation under oxidative and inflammatory stress conditions more effectively than ALDH^lo^ myoblasts.

ALDH^hi^ and ALDH^lo^ murine myoblasts were injected intramuscularly into the gastrocnemius of *mdx* mice to determine the degree of muscle regeneration in vivo. Muscles were excised and frozen for immunohistochemical characterization after a period of 14 days post-transplantation. The cell transplantations yielded regeneration of dystrophin (DYS) expressing myofibers (Figure 2h,i). The extent of myofiber formation was quantified using the regeneration index (RI) metric, a measure of DYS^+^ myofibers in cryosectioned *mdx* muscle per 10^5^ cells injected, as described previously [9]. Significantly greater RIs were observed in the muscles injected with ALDH^hi^ myoblasts compared to ALDH^lo^ myoblasts, suggesting that the regeneration index observed in vivo correlates with the stress resistance results obtained in vitro.

To further characterize the increased stress resistance capacity of the ALDH^hi^ myoblasts, we examined two major intracellular antioxidants, GSH and SOD. Significantly increased concentrations of GSH and increased activity of SOD was observed in ALDH^hi^ myoblasts compared to ALDH^lo^ myoblasts using spectrophotometric assays (Figure 2j,k). The association of elevated ALDH activity with elevated intracellular antioxidant levels suggests a potential mechanism by which these ALDH^hi^ cells are able to better withstand oxidative and inflammatory stress conditions in vitro and after implantation in vivo when compared to their ALDH^lo^ counterparts.

### The role of antioxidants and the ALDH enzyme in the stress resistance of ALDH^hi^ murine myoblasts

To further elucidate the role of intracellular antioxidant levels in the stress resistance capacity of ALDH sorted myoblasts, we altered the cells antioxidant levels prior to oxidative stress challenge. ALDH^lo^ myoblasts were treated with a mitochondrial targeted antioxidant, XJB-5-131 (XJB) prior to exposure to hydrogen peroxide to determine whether the proliferation rate could be elevated to that of ALDH^hi^ cells [56]. On the other hand ALDH^hi^ myoblasts were treated with a GSH sequestrator, DEM, prior to oxidative stress exposure in order to alter their intracellular antioxidant levels [57].

**Figure 3:**
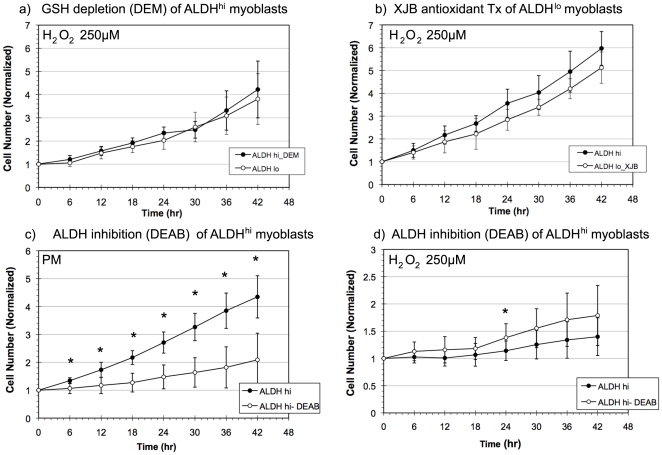
The role of antioxidants and ALDH in the stress resistance of murine ALDH^hi^ myoblasts. (a) When the antioxidant levels of ALDH^hi^ myoblasts were decreased by treatment with diethyl maleate (DEM), their proliferation rate was decreased to that of ALDH^lo^ myoblasts (n = 5). (* indicates p<0.05 at a given timepoint.) (b) By increasing the antioxidant levels of ALDH^lo^ cells with XJB prior to oxidative stress (H_2_O_2_, 250 µM) exposure, the proliferation rate in oxidative stress was increased to the levels observed in ALDH^hi^ myoblasts. (c) DEAB (50 µM) treatment of ALDH^hi^ murine myoblasts significantly decreased their proliferative rate in the absence of oxidative stress compared to untreated ALDH^hi^ myoblasts. (d) However, DEAB treatment had no statistically significant effect on the oxidative stress (H_2_O_2_, 250 µM) resistance of ALDH^hi^ myoblasts in terms of proliferation with the exception of an isolated data point at 24 hrs (p = 0.034).

Alteration of the antioxidant levels of these ALDH subpopulations had a significant impact on their proliferation capacities in oxidative stress. In contrast to the decreased proliferative capacity of ALDH^lo^ murine myoblasts compared to ALDH^hi^ myoblasts seen in Figure 2b, when the antioxidant levels of ALDH^lo^ cells were increased with XJB (500 nM) prior to oxidative stress exposure, the proliferation rate was significantly increased, to a level statistically equivalent to that observed in the ALDH^hi^ myoblasts (Figure 3b). On the other hand, when the antioxidant levels of ALDH^hi^ myoblasts were decreased by treatment with DEM (50 µM), the proliferation rate was significantly decreased to that observed in the ALDH^lo^ myoblasts (Figure 3a). This result suggests that the differences in stress resistance between the ALDH^hi^ and ALDH^lo^ myoblasts is conferred by differences in their oxidative stress handling capacities, which can be readily modified using antioxidant and pro-oxidant treatment.

Given the role of antioxidant activity in the stress resistance of ALDH^hi^ myoblasts, we questioned whether the ALDH enzyme was directly participating in the oxidative stress resistance of ALDH^hi^ myoblasts. ALDH has been directly implicated in the mitigation of oxidative damage by converting aldehyde by-products of lipid peroxidation such as malenaldehyde to non-reactive carboxylic acids [39], [41]. Furthermore, in the course of this reaction, the cofactor NADP^+^ is reduced to NADPH, which in turn may be oxidized in the process of GSH recycling via glutathione reductase. We hypothesized that ALDH activity may be directly responsible for the elevated oxidative stress resistance of ALDH^hi^ myoblasts, as found by Jean et al. [37], [58]. However, when we treated the ALDH^hi^ cells with DEAB (50 µM), a potent inhibitor of ALDH, we observed that DEAB impaired ALDH^hi^ myoblast proliferation in the absence of oxidative stress (Figure 3c), yet had no effect on the proliferation rate of ALDH^hi^ cells in oxidative stress conditions (250 µM H_2_O_2_) except at a single time point, 24 hrs (Figure 3d). This result suggests that ALDH antioxidant activity is not essential for the increased antioxidant capacity of murine ALDH^hi^ myoblasts.

### Increased chondrogenic and osteogenic differentiation potential of ALDH^hi^ myoblasts

It is well known that RA, a product of ALDH mediated oxidation of retinal, is required for embryonic myogenesis and can accelerate differentiation in postnatal derived myoblasts [27], [59]. RA works synergistically with bone morphogenic protein to promote osteogenesis, however its role in chondrogenesis is primarily inhibitory although it may be required at specific stages of differentiation [60], [61], [62], [63]. However, one may also consider the media conditions that are required to induce these differentiation pathways are deleterious to cells and represent a form of stress. For example one may consider the low serum conditions of myogenic differentiation medium as another kind of stress condition, which has been shown to induce apoptosis in those myoblasts that fail to upregulate p21 and Rb [64], [65]. Perhaps the cells' capacity to survive and function normally in adverse or altered media conditions is a necessary precondition to differentiate. We therefore hypothesized that elevated ALDH expression and stress resistance capacity could also favor osteogenic and chondrogenic differentiation in vitro.

**Figure 4:**
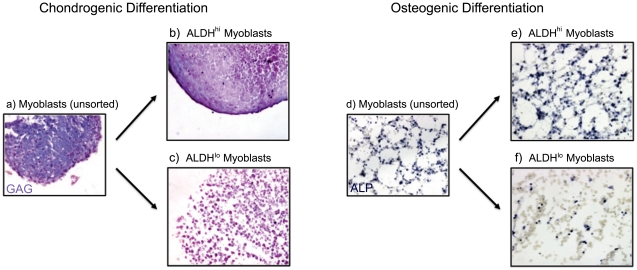
Chondrogenic and osteogenic potential of ALDH^hi^ myoblasts. (a–c) Chondrogenic differentiation in ALDH sorted myoblasts was induced via a pellet system containing TGF. Rapid proliferation and chondrogenic pellet formation was observed in ALDH^hi^ myoblasts (24 hrs) with robust production of glycosaminoglycans (GAGs), visualized via Alcian blue staining. This is in contrast to the poor proliferation and GAG production of ALDH^lo^ myoblasts. This result suggests that the chondrogenic differentiation capacity of unsorted myoblasts is dominated by ALDH^hi^ myoblasts. (d–f) Osteogenic differentiation of ALDH sorted myoblasts was induced in vitro by BMP-4 stimulation. Alkaline phosphatase levels (ALP, shown in blue), an early marker of osteogenic differentiation were significantly increased in ALDH^hi^ myoblasts compared to ALDH^lo^ myoblasts after a period of 4 d of BMP-4 stimulation.

We studied the chondrogenic and osteogenic differentiation of ALDH sorted myoblasts and found that, ALDH^hi^ myoblasts demonstrate a greater capacity to undergo chondrogenic and osteogenic differentiation in vitro when compared to ALDH^lo^ myoblasts (Figure 4). When chondrogenic differentiation was induced via a pellet system containing TGF-beta, rapid proliferation and chondrogenic pellet formation was observed in ALDH^hi^ myoblasts after 24 hrs with robust production glycosaminoglycans (GAGs, visualized via Alcian blue staining) (Figure 4b). This behavior mirrors that of the unsorted myoblasts (Figure 4a) suggesting that the chondrogenic potential of unsorted myoblasts may be conferred primarily by the ALDH^hi^ myoblasts (Figure 4b). However ALDH^lo^ myoblasts demonstrated poor proliferation in chondrogenic media and formed smaller, less dense pellets that required 2–3 d to coalesce (Figure 4c). Even after 21 d of culture, increased cell density and GAG formation was observed in ALDH^hi^ myoblasts compared to ALDH^lo^ myoblasts (Figure 4d–f).

Osteogenic differentiation of murine myoblasts was induced via bone morphogenic protein-4 (BMP-4) stimulation. The unsorted myoblast population increased their alkaline phosphatase expression, an early marker of osteogenic differentiation, in response to BMP-4 stimulation (Figure 4d), which was also observed in ALDH^hi^ myoblasts (Figure 4e). However a low alkaline phosphatase expression was observed in ALDH^lo^ myoblasts following BMP-4 stimulation (Figure 4f). As was observed in chondrogenic media conditions, the osteogenic differentiation potential of unsorted myoblasts appeared to be primarily due to the differentiation activity of ALDH^hi^ myoblasts (Figure 4d–f).

While the differences in osteogenic differentiation in ALDH sorted myoblasts may either be attributed to increased osteogenic capacity or increased media stress resistance, the case of chondrogenic differentiation capacity suggests the latter mechanism. It seems clear that ALDH^lo^ myoblasts experienced some form of growth retardation in chondrogenic media compared to ALDH^hi^ myoblasts to the extent that ALDH^lo^ myoblasts did not form a condensed pellet. Furthermore increased RA production by the ALDH^hi^ cell would not be expected to increase the chondrogenic differentiation capacity of these cells. The inability of ALDH^lo^ myoblasts to survive and proliferate in chondrogenic media impaired their capacity for chondrogenic differentiation, as measured by pellet size and amount of GAG production. Consequently, we believe that the increased proliferation potential of ALDH^hi^ myoblasts may not only improve the myogenic differentiation but also facilitate increased chondrogenic and osteogenic differentiation capacities.

### ALDH^hi^ and ALDH^lo^ sorted murine MDSCs do not display a difference in stress resistance, proliferation, differentiation, and muscle regeneration capacity

**Figure 5:**
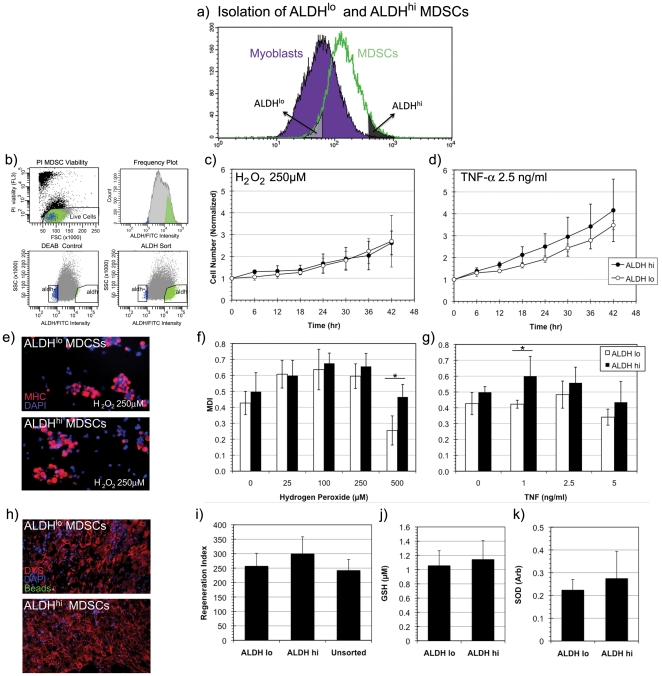
Isolation and stress resistance capacity of ALDH^hi^ MDSCs. (a) A schematic of the isolation of ALDH^lo^ and ALDH^hi^ subpopulations of MDSCs is depicted here. (b) As discussed previously, ALDH^lo^ and ALDH^hi^ MDSCs were isolated, by excluding propidium iodide stained dead cells, and forming appropriate ALDH gates using DEAB inhibited controls. (c, d) No statistically significant difference in proliferative capacity between ALDH^lo^ and ALDH^hi^ MDSCs in oxidative stress (H_2_O_2_, 250 µM) or in inflammatory stress (TNF-α, 2.5 ng/ml) was observed (n = 9). (e) ALDH sorted MDSCs underwent myogenic differentiation in oxidative stress (H2O2, 250 µM) by expressing myosin heavy chain (MHC) and fusing into MHC^+^ clusters (red) rather than fusiform myotubes. (f, g) Significant differences in myogenic differentiation indices (MDI) were not observed in ALDH^hi^ MDSCs compared to ALDH^lo^ MDSCs except at one very high oxidative stress (H_2_O_2_, 500 µM) and at an intermediate inflammatory stress level (TNF-α, 1 ng/ml) (n = 9,* indicates p<0.05). (h) Robust engraftment of ALDH^lo^ and ALDH^hi^ MDSCs was observed following intramuscular injection into the gastrocnemius of *mdx* mice. Dystrophin (DYS) positive myofibers (stained in red) indicate transplanted MDSC myofiber generation or fusion with a host myofiber. (i) No statistically significant differences in regeneration index were observed in ALDH^hi^ transplanted mice compared to those injected with ALDH^lo^ and unsorted MDSCs (n = 3). (j, k) Measurements of intracellular antioxidant levels in terms of glutathione (GSH) and superoxide dismutase (SOD) levels yielded no statistically significant differences between ALDH^lo^ and ALDH^hi^ MDSCs (n = 3).

Given the heterogeneity of ALDH activity and stress resistance in myoblasts, we hypothesized that such heterogeneity may not be observed in MDSC populations since we have previously reported that MDSCs are highly resistant to stress [9]. ALDH^lo^ and ALDH^hi^ subpopulations of murine MDSCs were isolated by FACS in the same manner as murine myoblasts as depicted in Figure 5a. A representative FACS isolation of ALDH^lo^ and ALDH^hi^ MDSCs is illustrated in Figure 5b.

In contrast to myoblasts, MDSCs did not display a high degree of heterogeneity in stress resistance behavior when sorted into ALDH^lo^ and ALDH^hi^ subpopulations. No significant differences in proliferation (Figure 5c, d) or myogenic differentiation (Figure 5e-g) capacity under conditions of oxidative and inflammatory stress were observed between ALDH^lo^ and ALDH^hi^ MDSCs. Although a perceptible trend of an increase in the myogenic differentiation index was observed in ALDH^hi^ MDSCs when compared to ALDH^lo^ MDSCs, these trends were not statistically significant except at one very high oxidative stress dose (H_2_O_2_, 500 µM) and one intermediate inflammatory stress dose (TNF-α, 1 ng/ml). While these trends suggest that there may be a slight difference in stress resistance between ALDH^hi^ and ALDH^lo^ MDSCs, these trends were not consistently statistically significant.

When injected intramuscularly into the *mdx* mouse gastrocnemius, both ALDH^lo^ and ALDH^hi^ MDSC subpopulations induced robust muscle regeneration and formation of numerous dystrophin expressing muscle fibers; however, no significant difference in the RIs of ALDH^lo^ and ALDH^hi^ MDSCs was observed (Figure 5h, i). Not surprisingly, there were no significant differences observed in the GSH or SOD levels between the two ALDH sorted subpopulations of MDSCs (Figure 5j, k). This result suggests that no obvious difference in stress resistance, proliferation, myogenic differentiation, or regenerative capacity in skeletal muscle exists between ALDH^lo^ and ALDH^hi^ subpopulations of MDSCs, a population of muscle cells endowed with a high resistance to stress [9].

### Increased stress resistance, proliferation, differentiation and muscle regeneration capacity of ALDH^hi^ sorted human muscle derived cells

**Figure 6:**
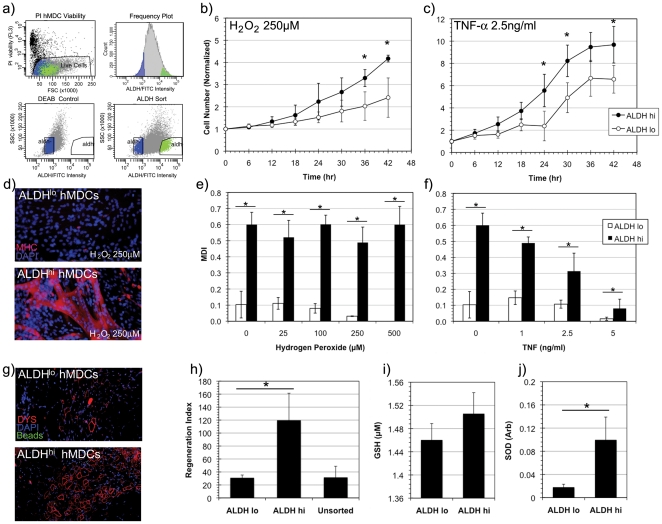
Isolation and stress resistance capacity of ALDH^hi^ human muscle derived cells (hMDCs). (a) ALDH^hi^ hMDCs were isolated using FACS. (b, c) Significantly increased rates of proliferation of ALDH^hi^ hMDCs compared to ALDH^lo^ hMDCs were observed in conditions of oxidative (H_2_O_2_, 250 µM) and inflammatory stress (TNF-α, 2.5 ng/ml) (n = 6, * indicates p<0.05). (d) Myogenic differentiation capacity was measured in low serum conditions by myosin heavy chain (MHC, shown in red) expression. ALDH^hi^ hMDCs generated dense networks of MHC^+^ myotubes in oxidative stress conditions (H_2_O_2_, 250 µM) in contrast to ALDH^lo^ hMDCs. (e, f) Significantly increased myogenic differentiation indices (MDI) were calculated in ALDH^hi^ hMDCs in oxidative (H_2_O_2_) and inflammatory (TNF-α) stress conditions compared to ALDH^lo^ hMDCs. (g, h) Dystrophin (DYS, red) positive myofiber regeneration was observed following intramuscular injection of ALDH sorted hMDCs. A significantly increased regeneration index was observed in ALDH^hi^ hMDC transplanted *mdx*/SCID mice compared to those injected with ALDH^lo^ and unsorted hMDCs (n = 3). (i) While a trend of increased GSH levels was observed between ALDH^hi^ and ALDH^lo^ hMDCs, this increase was not statistically significant. (j) However, significantly increased superoxide dismutase (SOD) activity was observed in ALDH^hi^ hMDCs when compared to ALDH^lo^ hMDCs (n = 3).

Given the clinical relevance of working with human muscle derived cells, we proceeded to study unfractionated or primary hMDCs to determine whether their ALDH sorted subpopulations would exhibit similar behavior to murine muscle derived cells. We sought to identify a similar ALDH^hi^ subpopulation of cells from cultured human primary cells using the same methodology described for murine myoblasts and MDSCs (Figure 6a). Indeed, the behavior of hMDCs sorted on the basis of ALDH activity demonstrated many similarities to murine myoblasts in terms of differences in stress resistance in vitro as well as regeneration indices in vivo.

A dramatic increase in oxidative and inflammatory stress resistance was observed in terms of proliferation and myogenic differentiation in ALDH^hi^ hMDCs compared to ALDH^lo^ hMDCs. A significant proliferation advantage of ALDH^hi^ hMDCs compared to ALDH^lo^ hMDCs was observed (Figure 6b, c) when exposed to either oxidative or inflammatory stress conditions. Similarly, a significantly increased MDI was observed in ALDH^hi^ hMDCs compared to ALDH^lo^ hMDCs at all oxidative and inflammatory stress doses.

In vivo, a significantly increased RI was observed in the gastrocnemius muscles of *mdx*/SCID mice transplanted with ALDH^hi^ hMDCs compared to those injected with ALDH^lo^ and unsorted hMDCs (Figure 6g-h). As in the case of ALDH sorted murine myoblasts, we observed a strong correlation between stress resistance in vitro with regeneration capacity in vivo.

To determine if the increased stress resistance capacity of the ALDH^hi^ hMDCs may be conferred by elevated intracellular antioxidants like ALDH^hi^ myoblasts, we examined the GSH and SOD levels in ALDH^lo^ and ALDH^hi^ hMDCs. Although a trend of increased GSH was observed in ALDH^hi^ hMDCs compared to ALDH^lo^ hMDCs, this trend was not statistically significant (Figure 6i). However a significant difference in intracellular SOD was observed between the ALDH^hi^ and ALDH^lo^ hMDCs (Figure 6j). Again, an association of elevated ALDH activity with elevated intracellular antioxidant levels suggests a mechanism by which these cells are able to withstand oxidative and inflammatory stress conditions, above that which may be conferred by ALDH itself.

## Discussion

Our research on the muscle regenerative capacity of various populations of myogenic progenitors suggests that stress resistance is predictive of muscle regeneration capacity [9], [10], [11]. Following transplantation, precipitous and acute cell loss is observed which has been attributed to inflammation, anoikis, and ischemia [66]. The conditions that donor cells encounter upon transplantation are deleterious to survival and tissue specific differentiation, due to the host inflammatory process initiated within minutes of transplantation. This exposes the cells to oxidants such as H_2_O_2_, cytotoxic radicals, and pro-inflammatory cytokines [67]. In the 24–48 hrs following transplantation, cell competence in terms of survival, proliferation, and myogenic differentiation largely determines efficacy of subsequent skeletal muscle regeneration. We have shown that by modulating the antioxidant levels of MDSCs we can alter its skeletal muscle regenerative capacity both in cardiac and skeletal muscle [9], [11]. Similar studies using antioxidant and anti-inflammatory treatments such as SOD-mimetics [68], anti-TNF-α, and anti-neutrophil antibodies [5], [69] further demonstrate the importance of stress resistance in the context of cell survival in cell therapy. While numerous immunosuppressive and antioxidant modalities have been identified to increase cell survival following transplantation, cellular candidates for transplantation may also be screened for or isolated by their inherent survival capabilities [66], [70]. It is this approach that has motivated the present study as well as other ongoing studies in our group.

### The role of ALDH on the stress resistance and differentiation capacity

In the current study, ALDH^hi^ muscle progenitor cells isolated by FACS from murine and human skeletal muscle demonstrated an enhancement in cell proliferation and myogenic differentiation capacities, when cultivated in conditions of oxidative (H_2_O_2_) and inflammatory stress (TNF-α). Vauchez et al. and Jean et al. have previously isolated ALDH^hi^ progenitors from human muscle [16], [37]. However, Jean et al. found ALDH^hi^ cells to be absent in murine muscle, despite the isolation of ALDH^hi^ cells from other murine tissues by others [37], [71]. The source of the difference in our observations is not entirely clear, however it may lie in part in differences our isolation protocols. Jean et al. studied the cells isolated by FACS immediately following pronase digestion of murine paw muscles; however, the age and sex of their animals were not cited [37]. Our use of muscle from neonatal female mice, which we have found to be enriched with stem cells, and our use of the preplate technique prior to FACS isolation may account for the difference in our observations [12], [37].

While ALDH has been shown to mitigate oxidative damage such as lipid peroxidation, our results indicate that increased intracellular antioxidant levels observed in ALDH^hi^ cells are primarily responsible for the increased oxidative stress resistance. This conclusion is not entirely consistent with the findings of Jean et al. who observed a dramatic loss in viability of human ALDH^hi^ myoblasts in oxidative stress when ALDH activity was inhibited by DEAB [37]. However we observed no loss in proliferative capacity when murine ALDH^hi^ myoblasts were treated with DEAB. The difference in our observations however likely stems from differences in our experimental design. Jean et al. used survival as their metric of oxidative stress resistance, whereas we measured proliferation, which clearly employs different cellular pathways and may have a different sensitivity of oxidative stress. Jean et al. observed differences in survival at H_2_O_2_ concentrations of 400–650 µM, far greater than the concentration we required (250 µM) to observe differences in proliferation. We concluded that ALDH activity likely plays a role but does not represent a major determinant in the oxidative stress capacity of ALDH^hi^ cells. However, increased reservoirs of intracellular antioxidants such as GSH and SOD in murine and human ALDH^hi^ murine myoblasts and hMDCs appear to have a greater impact on oxidative stress resistance than ALDH activity.

ALDH mediated production RA has been shown to promote myogenic differentiation but play a more nuanced or synergistic role in osteogenic and chondrogenic differentiation [59], [62], [63], [72], [73], [74]. The increased myogenic differentiation capacity of both ALDH^hi^ murine myoblasts and hMDCs in the absence of H_2_O_2_ and TNF-α suggests an inherent elevated myogenic capacity compared to their ALDH^lo^ counterparts, clearly implicating the RA producing activity of ALDH. These results are corroborated by those of Vauchez et al., who observed increased myogenic capacity of ALDH^hi^ human myoblasts [16]. However, elevated RA cannot account for the preservation of this myogenic capacity at increasing stress doses observed in our paper. This must be mediated, again, by the increased antioxidant capacity of ALDH^hi^ cells as discussed previously. This conclusion is reinforced by the observation that ALDH^lo^ myoblasts, while capable of myogenic differentiation, lose this capacity rapidly with increasing stress doses.

RA has been shown to promote osteogenesis synergistically with bone morphogenic protein [61], [73]. This suggests that ALDH^hi^ myoblasts may be expected to have increased osteogenic differentiation compared to ALDH^lo^ myoblasts when treated with BMP-4 as we observed in our murine muscle derived cells. Vauchez et al. also observed osteogenic differentiation in their ALDH^hi^ human cells however they did not compare this capacity to their ALDH^lo^ cells [16]. Although some authors have shown RA to impair chondrogenesis others have shown that it is required for chondrogenesis but only at certain points in chondrogenic differentiation [62], [63] Therefore one would not necessarily expect ALDH^hi^ cells to undergo chondrogenesis at an enhanced rate compared to ALDH^lo^ cells. However, we observed not only increased proliferation in chondrogenic media but also increased GAG production by ALDH^hi^ myoblasts. In this case the RA does not necessarily have a role in promoting chondrogenesis and almost certainly does not have a role in promoting proliferation in this setting. We posit that this result may simply be explained by an increased capacity of these cells to survive the chondrogenic and osteogenic media conditions, given the poor proliferation of ALDH^lo^ myoblasts in chondrogenic media and inability to form a dense chondrogenic pellet.

### ALDH activity in MDSCs: Implications on the preplate technique

We observed an enrichment of ALDH^hi^ cells in late preplate cells (slowly adhering cells), a population enriched with MDSCs which exhibit elevated oxidative stress resistance [9]. It is possible that the preplate technique segregates cells based on their ability to remain viable in suspension and resist detachment induced apoptosis or anoikis [75]. Given our observation, of an enrichment of SACs in ALDH^hi^ murine muscle derived cells (Figure 1e), perhaps the enhanced survival of these cells includes increased resistance to anoikis. These results suggest that the yield of the preplate technique could be improved or the process shortened to fewer preplate cycles if it is preceded by FACS isolation of ALDH^hi^ cells.

No improvement in stress resistance was observed in ALDH^hi^ MDSCs compared to ALDH^lo^ MDSCs in terms of proliferation and myogenic differentiation. This result is not surprising given the increased homogeneity of MDSCs in addition to previous observations of increased stress resistances in MDSCs in general compared to myoblasts [9]. These experiments demonstrate that the stress resistance of MDSCs is not further enhanced by isolating an ALDH^hi^ sub-population.

### Implications of in vitro behavior on muscle regeneration in vivo

The improved proliferative capacity of murine ALDH^hi^ myoblasts and hMDCs over that of their ALDH^lo^ counterparts, in the presence of H_2_O_2_ and TNF-α in vitro suggests that the ALDH^hi^ cells may have an increased proliferative capacity under conditions of post-transplantation inflammation in vivo. Similarly, an increased differentiation capacity in conditions of oxidative and inflammatory stress is likely to translate to an improvement in the differentiation capacity when the cells are exposed to post-transplantation inflammation. Indeed we observed improved muscle regeneration in muscles injected with both murine or human ALDH^hi^ muscle cells compared to their ALDH^lo^ counterparts, which appears to be a function of improved survival, proliferation, and myogenic differentiation.

The source of this improved regeneration capacity of ALDH^hi^ myogenic cells may be increased stress resistance, due to the elevated intracellular levels of antioxidants such as GSH and SOD in addition to the stress resistance role of ALDH, an observation that was also made previously in MDSCs [9]. The finding that increasing the antioxidant levels of ALDH^lo^ myoblasts by treating them with XJB improved their proliferative capacity to a similar level observed in the ALDH^hi^ myoblasts supports this hypothesis. Furthermore, decreasing the GSH antioxidant levels of ALDH^hi^ myoblasts via DEM treatment inhibited their proliferative capacity to a similar level observed in the ALDH^lo^ myoblasts.

While numerous myogenic cells have been identified for muscle cell therapies, identification of definitive or even desirable markers for myogenic progenitor isolation remains elusive [5]. In our experience, a common feature of myogenic progenitors with high regeneration potential lies in their increased resistance to oxidative and inflammatory stress [9], [10]. In the current study, we isolated ALDH^hi^ and ALDH^lo^ subpopulations of cultured murine myoblasts and MDSCs in addition to hMDCs. The utility of isolating myogenic cells with elevated ALDH expression is two-fold. Myogenic progenitors may be rapidly isolated from heterogeneous populations of muscle derived cells using a simple intracellular dye, Aldefluor. In addition, ALDH may be used as a marker to identify cells with an increased proliferative and differentiation capacity when exposed to oxidative and inflammatory stress.

Although it has been known for many years that stem and progenitor cells display unique behaviors that are advantageous to applications of cell therapy such as self-renewal, long-term proliferation, and multipotent differentiation, our results suggest that resistance to stress should be included among these vital attributes. Stem cell behavior such as improved survival may even represent a more important “marker” for stem cell therapy than the use of traditional surface marker profiles. This particular hypothesis is supported by our extensive studies of MDSCs, which are isolated by their slow adhesion behavior and their remarkable survival capacity in vitro and in vivo [9], [17], [76], [77]. In our continued work, we seek a greater understanding of the mechanisms responsible for increased regeneration capacity and an understanding of what allows some progenitors greater survival abilities in the presence of oxidative and pro-inflammatory environments. The data presented in this study suggests that ALDH can be used as a marker to identify progenitors that are not only myogenic but have elevated oxidative and inflammatory stress resistance.

## Materials and Methods

### Animal usage

All animal procedures were performed in accordance with the Guide for the Care and Use of Laboratory Animals (NIH Publications 85-23) as promulgated by the Committee of Care and Use of Laboratory Animals of the Institute of Laboratory Sciences, the National Academy of Sciences, and the National Research Council. The University of Pittsburgh's Institutional Care and Use Committee (IACUC) reviewed and approved all the procedures performed in these studies (IACUC protocol #: 0902596A-2).

### Murine cell isolation

Murine myoblasts and MDSCs were isolated from the skeletal muscle of 3-wk-old C57BL/6J female mice (The Jackson Laboratory, Bar Harbor, ME) as previously described using a modified preplate technique [12], [17]. Muscle extracted from hindlimbs were minced into a slurry and enzymatically dissociated at 37°C in 0.2% collagenase-type XI (Sigma-Aldrich) for 1 hr. The cells were then incubated in dispase (2.4 U/ml HBSS, GIBCO, Invitrogen) for 45 min then for 30 min in trypsin-EDTA (0.1% in HBSS, GIBCO, Invitrogen). The dissociated cells were passed through a 70 µm filter, centrifuged and resuspended in proliferation medium (PM) to initiate the preplate process. PM consists of 10% horse serum (GIBCO), 10% FBS (GIBCO), 0.5% chick embryo extract (Accurate Chemical Company), and 1% penicillin–streptomycin (GIBCO) in DMEM high glucose (Invitrogen).

The preplate technique segregates muscle derived cell populations by how quickly they adhere to collagen-coated plates as described elsewhere [12], [76]. Briefly, cells are plated on collagen-I coated plates for a predetermined amount of time (PP1: 2 hrs, PP2-PP6: 24 hrs) at which point non-adherent cells are collected, centrifuged and plated for the next preplate cycle, as depicted in Figure S1a. Each preplate population has been previously characterized, such that each subsequent preplate cycle is enriched with myogenic, desmin-positive cells [50], [78], [79]. MDSCs are isolated from long-term proliferating colonies of PP6.

Myoblasts were isolated from rapidly adhering cell fractions of the early preplate cycles while MDSCs were isolated from the slowly adhering cell fraction of the late preplate cells (PP6). All cells were cultured and expanded in PM in collagen-coated flasks at 37°C and 5% CO_2_ as described previously [12]. When applying the preplate technique to ALDH sorted cells (as shown in Figure 1e), the digestion process was identical to that described above; however, the digested muscle cells were sorted for ALDH activity using FACS prior to performing the preplate protocol.

### Human muscle cell isolation

Human gastrocnemius muscle biopsies, 9 in total, were obtained (mean age 58 years; range 25 to 75 years, both male and female) from the National Disease Research Interchange (NDRI) and the Center for Organ Recovery and Education (CORE). All biopsies were taken from patients with no history of neuromuscular/skeletal disease, sepsis, chemotherapy, drug abuse, or ventilatory support greater than 1 month. Furthermore, NDRI and CORE obtain written informed consent from the patient's family prior to harvesting any organs or tissues. Acquisition of muscle from the NDRI and CORE, and subsequent muscle stem cell isolation, was performed in accordance to the protocol reviewed and approved by the University of Pittsburgh's Institutional Review Board (Protocol #: PRO09030265, entitled: Isolation of Human Muscle-Derived Stem Cells). Tissue was finely minced then digested for 60 min at 37°C with 2.4 U/mL dispase (GIBCO), type-I and type-IV collagenases (both at 0.5 mg/ml; Sigma-Aldrich). The digested tissue was pelleted and resuspended in DMEM supplemented with 10% fetal bovine serum (FBS) and 1% penicillin/streptomycin (P/S), then passed through a 100 µm filter followed by a 70 µm filter to obtain a single cell suspension. Cells were then treated with red cell lysis buffer for 15 minutes before being cultured in PM in collagen-I coated flasks at 37°C and 5% CO_2_. PM consists of 10% horse serum (GIBCO), 10% FBS (GIBCO), 1% chick embryo extract (Accurate Chemical Company), and 1% penicillin–streptomycin (GIBCO) in DMEM high glucose (Invitrogen). Following enzymatic dissociation, human muscle cells were cultured for three to four days in the flasks of their original seeding and were passaged one to two times as necessary prior to FACS. This protocol is illustrated in Figure S1b. It should be noted that these hMDCs were almost uniformly CD56 positive, indicating their myogenic lineage (data not shown).

### Fluorescence activated cell sorting by ALDH activity

Cultured murine and human cells were trypsinized, washed in cold PBS, and counted using a hemocytometer. Cells (10^6^) of each population were resuspended in Aldefluor buffer, which contains an ABC transport inhibitor that prevents efflux of the Aldefluor dye, and incubated at 37°C with BAAA according to the manufacturer's instructions (Aldagen Inc, Durham, NC). Cells were washed in Aldefluor buffer and maintained in 4°C throughout the cell sorting process. ALDH activity was assessed using the FL1 channel of a BD FACSAria Cell Sorting System and FACSDiva software (version 6.1.2) (Becton, Dickinson and Company, San Jose, CA). Collected cells were gated on their fluorescence intensity, which corresponds to their ALDH activity levels, as well as low side scatter (SCC^lo^). Sorted cells were recaptured in cold (4°C) PM and immediately plated in collagen-I coated flasks and normal incubation conditions (5% CO_2_ at 37°C).

### In vitro proliferation capacity

Time-lapse live cell microscopy was employed to monitor the rate of proliferation under conditions of oxidative and inflammatory stress [80], [81]. Sorted human muscle derived cells were plated in collagen-I coated 24-well plates at an initial density of 1000 cells/cm^2^ in PM and incubated under normal conditions for 24 hrs. The media was exchanged with PM treated to simulate oxidative (250 µM, H_2_O_2_, Sigma-Aldrich) and inflammatory stress (2.5 ng/ml, TNF-α, both mouse and human recombinant, R&D Systems) and immediately placed in a live cell imager. Cells were imaged at 30 min intervals in three 10× microscope fields per well over a 48 hr time period under normal incubation conditions (5% CO_2_ at 37°C).

### In vitro muscle differentiation capacity

The capacity of ALDH sorted cell populations to differentiate into MHC expressing cells under varying conditions of oxidative and inflammatory stress was quantified in vitro using the MDI metric. Cells were plated at 1000 cells/cm^2^ in 24-well plates for 2 d in PM or until near confluence. PM was then exchanged for differentiation media (DM; 2% HS, 1% P/S in DMEM) treated to simulate oxidative stress (25 µM, 100 µM, 250 µM, and 500 µM, H_2_O_2_, Sigma-Aldrich) and inflammatory stress (1 ng/ml, 2.5 ng/ml, and 5 ng/ml, TNF-α, R&D Systems). The media was exchanged daily to maintain constant stress levels [9]. Cells were allowed to differentiate and fuse for 4 d then fixed with cold methanol (−20°C). The MDI was quantified via immunohistochemical staining for MHC expression as described in “Immunohistochemistry” methods section.

### Antioxidant capacity

The antioxidant capacity was measured in terms of the activity of the major intracellular antioxidant molecules: GSH and SOD. Levels of GSH were measured colorimetrically (Calbiochem, 354102) using a spectrophotometer (TECAN Infinite M200, Männedorf, Switzerland). GSH detection is mediated by capture of all thiol mercaptans (RSH) from mechanically homogenized cells into thioethers by 4-chloro-1-methyl-7-trifluromethyl-quinolinium methylsulfate followed by the formation of a chromophoric thione in those GSH specific thioethers using NaOH. GSH levels were quantified by chromophoric thione absorbance at 400 nm. Total activity of SOD was measured using a colorimetric assay (Chemicon, Temecula, CA; APT290). SOD levels of chemically lysed cells (10 mM Tris, pH 7.5, 150 mM NaCl, 0.1 mM EDTA, and 0.5% Triton X-100) were measured using a xanthine/xanthine oxidase system in which superoxide-chromagen absorbance (490 nm) is lowered by the presence of SOD.

The intracellular antioxidant levels of ALDH^lo^ and ALDH^hi^ myoblasts were modified by a chemical antioxidant (XJB-5-131, generously donated by Prof. Peter Wipf at the University of Pittsburgh) and DEM, a pro-oxidant (Sigma-Aldrich). XJB is a membrane permeable, mitochondrial targeted nitroxide antioxidant that has proven to be cytoprotective in disease states associated with oxidative stress, such as hemorrhagic shock, and mitigates apoptotic cell death in vitro [82], [83]; whereas DEM, is a chemical that conjugates and inactivates GSH thus decreasing the cell's capacity to resist oxidative stress [84]. To examine the role of antioxidant levels in ALDH sorted myoblasts, ALDH^lo^ myoblasts were pretreated with 500 nM XJB in PM for 2 hrs prior to exposure to oxidative stress (250 µM H_2_O_2_ in PM) conditions. ALDH^hi^ myoblasts were pretreated with 50 µM DEM for 2 hrs prior to exposure to oxidative stress (250 µM H_2_O_2_ in PM) conditions. In both cases the myoblasts were washed in PBS prior to stress exposure to insure all active antioxidants or pro-oxidants were intracellular and not in the media. The rates of proliferation and survival of these pretreated myoblasts were quantified using a live cell imager (Kairos Instruments LLC, Pittsburgh, PA) and compared to that of non-pretreated controls in identical oxidative stress conditions.

To examine the role of ALDH in the antioxidant capacity of ALDH sorted myoblasts a potent ALDH inhibitor, DEAB, was used. Pretreatment of ALDH^hi^ myoblasts with 50 µM of DEAB in PM was used 24 hrs prior to oxidative stress exposure. A concentration of 50 µM DEAB was maintained during the oxidative stress exposure to maintain ALDH inhibition throughout the experiment. The rates of proliferation and survival were quantified using a live cell imager and ImageJ software (NIH).

### Skeletal muscle regeneration capacity

Skeletal muscle regeneration capacity of ALDH sorted myogenic cells was quantified using the regeneration index metric as previously described [9], [17], [51]. Briefly, the number of dystrophin positive myofibers per cryosection per 10^5^ cells injected is recorded. ALDH sorted murine cells were injected into the gastrocnemius muscles of female *mdx* mice (C57BL/10ScSn-DMD^mdx^/J, The Jackson Laboratory), which is a murine model of Duchenne muscular dystrophy using a protocol previously described [9], [85]. The *mdx* mouse is a C57BL strain homozygous for a spontaneous X-linked mutation of the dystrophin gene and lacks dystrophin protein expression [85]. Animals were sacrificed 14 d following injection. ALDH sorted human muscle cells were injected into the gastrocnemius muscles of male *mdx*/SCID mice (C57BL/10ScSn-DMD^mdx^-SCID/J, The Jackson Laboratory) aged 6–8 weeks using a protocol previously described [10]. Cells, cultured in T-175 collagen-I coated flasks, were trypsinized, counted, washed in cold PBS, and resuspended in a cold PBS suspension of FluoSpheres (Molecular Probes), at a concentration of 2×10^6^ cells per 20 µl, prior to injection. Animals were sacrificed at 10 d after injection to conform to previous human cell transplantation experiments in our lab [10]. All gastrocnemius muscles were excised and frozen in 2-methyl-butane, cryosectioned (8 µm), and mounted on glass slides.

### Immunohistochemistry

Differentiation of myogenic cells into MHC expressing cells in vitro was quantified using the MDI metric described previously. Briefly, samples were blocked with 5% HS and incubated with monoclonal antibodies for MHC (Sigma; 1∶300) followed by Cy3-conjugated anti-mouse antibodies. Nuclei were stained blue with 4, 6-diamidino-2-phenylindole (DAPI, Sigma; 100 ng/ml, 1∶1000). At least 200 cell nuclei were counted for each MDI measurement.

All murine skeletal muscle tissue samples were frozen in 2-methylbutane cooled in liquid nitrogen, then stored at –80°C. 8 µm cryosections were fixed in 5% formalin for 2 min and blocked in 5% donkey serum for 1h. Skeletal muscle sections were stained for dystrophin (DYS) using an anti-dystrophin primary antibody (1∶300, Rabbit Anti-DYS, Abcam Ab15277) and a secondary anti-rabbit antibody (1∶500, Donkey anti-Rabbit, Molecular Probes A21207) using a protocol previously described [86]. Nuclei were stained with DAPI. Fluorescence and brightfield microscopy was performed using a Nikon Eclipse E800 microscope (Melville, NY) equipped with a Retiga Exi digital camera (QImaging) and Olympus FV 500 confocal scanning microscope. All images were acquired and analyzed using Northern Eclipse software (version 6.0; Empix Imaging).

### Osteogenic and chondrogenic differentiation

ALDH^hi^, ALDH^lo^ and unsorted cells were prepared in pellet form (2.5×10^5^ cells) or as a monolayer (1.5×10^4^ cells/well in 12 well plates). For osteogenesis, murine myoblasts were plated on collagen-I coated 12 well plates and maintained for 4 d in osteogenic media (Lonza, Walkersville, MD), which included dexamethasone, glutamine, ascorbate, penicillin/streptomycin (P/S), β-glycerophosphate and bone morphogenic protein-4 (50 ng/ml). The cells were stained for alkaline phosphatase following the manufacturer's instructions (Sigma-Aldrich, 86C-1KT). Chondrogenesis of murine myoblasts was studied by culturing the cells in a pellet culture by incubating the cell pellets for 21 d in chondrogenic media (Lonza, PT3003), which contains dexamethasone, ascorbate, ITS+, P/S, sodium pyruvate, proline, glutamine and TGF-beta-3 (10 ng/ml) (Lonza, PT4124). All pellets were fixed in 10% formalin for 24 hrs followed by dehydration, paraffin embedding, sectioning, and Alcian blue staining.

### Statistical Analysis

Data are expressed as a mean with its standard deviation. Direct comparisons between two cell populations were made using an unpaired, two-tailed Student's *t*-test, where p<0.05 was considered to be statistically significant. Comparisons of multiple groups were completed using one-way ANOVA followed by Tukey post-hoc comparisons.

## Supporting Information

Figure S1 **Isolation diagram of murine and human ALDH sorted cells.** (a) Murine myoblasts and MDSCs were isolated by a modified preplate technique, as described previously. ALDH^hi^ and ALDH^lo^ subpopulations of these muscle derived cells were isolated by FACS for subsequent expansion in proliferation medium. (b) hMDCs were isolated by enzymatic digestion of human skeletal muscle. Following 3-4 d of culture, ALDH^hi^ and ALDH^lo^ subpopulations of hMDCs were isolated by FACS for subsequent expansion in proliferation medium.
(TIF)

Table S1 **Data summary of ALDH sorted populations isolated from skeletal muscle.**
(TIFF)
