# Supplementary figures and images for: Murine and Human Myogenic Cells Identified by Elevated Aldehyde Dehydrogenase Activity: Implications for Muscle Regeneration and Repair
Source: PLoS One. 2011 Dec 15;6(12):e29226. doi: 10.1371/journal.pone.0029226 (PMC3240661; doi:10.1371/journal.pone.0029226)

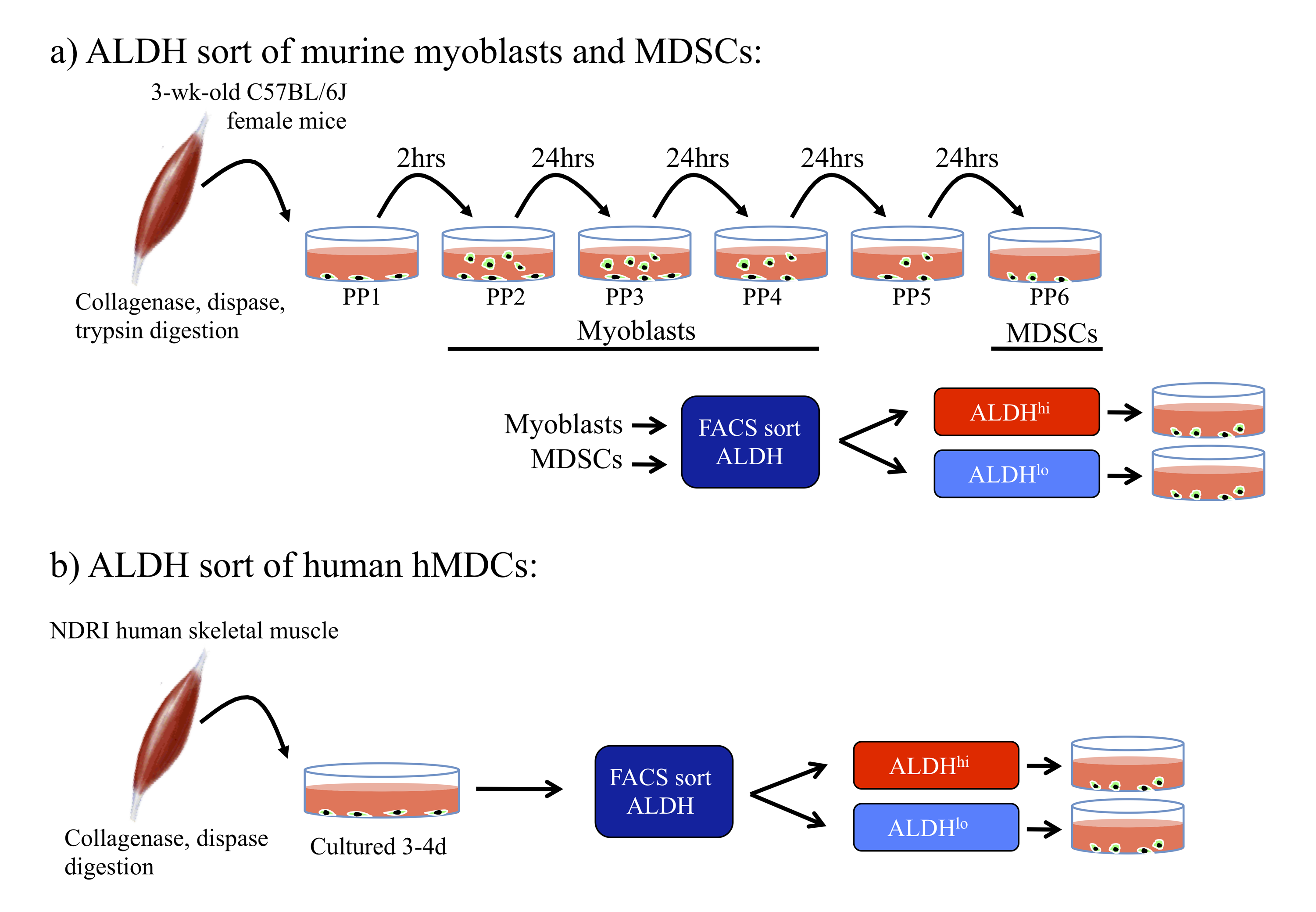

Supplement: Figure S1 — Isolation diagram of murine and human ALDH sorted cells. (a) Murine myoblasts and MDSCs were isolated by a modified preplate technique, as described previously. ALDHhi and ALDHlo subpopulations of these muscle derived cells were isolated by FACS for subsequent expansion in proliferation medium. (b) hMDCs were isolated by enzymatic digestion of human skeletal muscle. Following 3-4 d of culture, ALDHhi and ALDHlo subpopulations of hMDCs were isolated by FACS for subsequent expansion in proliferation medium. (TIF) [file pone.0029226.s001.tif]

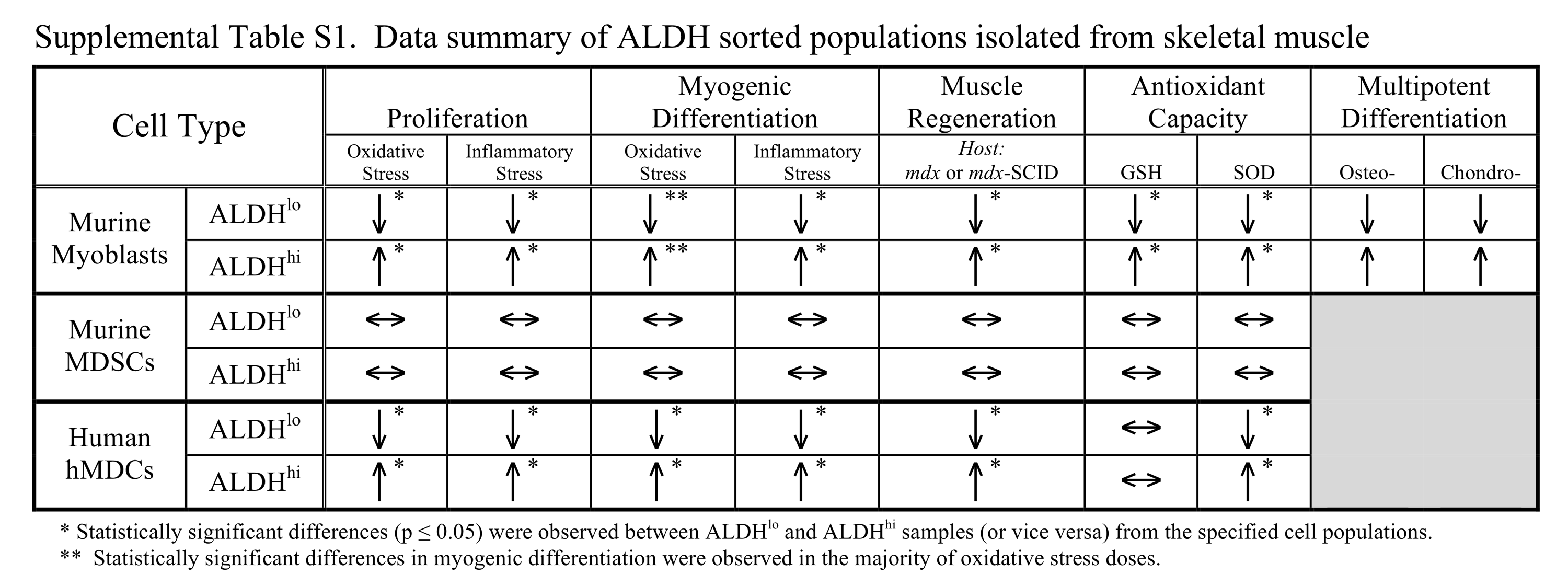

Supplement: Table S1 — Data summary of ALDH sorted populations isolated from skeletal muscle. (TIFF) [file pone.0029226.s002.tiff]
